# From tradition to precision: leveraging digital tools to improve cattle health and welfare

**DOI:** 10.3389/fvets.2025.1549512

**Published:** 2025-04-02

**Authors:** Andra-Sabina Neculai-Valeanu, Catalina Sanduleanu, Ioana Porosnicu

**Affiliations:** ^1^Laboratory of Nutrition, Quality and Food Safety, Department of Research, Research and Development Station for Cattle Breeding, Iasi, Romania; ^2^The Academy of Romanian Scientists, Bucharest, Romania; ^3^Department of Animal Resources and Technologies, Faculty of Food and Animal Resources, Iasi University of Life Science, Iasi, Romania; ^4^Department of Public Health, Faculty of Veterinary Medicine, Iasi University of Life Science, Iasi, Romania

**Keywords:** digital revolution, cattle health, welfare, precision livestock farming, internet of things, artificial intelligence, predictive technologies, monitoring systems

## Abstract

Traditional cattle production practices relied heavily on manual observation and empirical decision-making, often leading to inconsistent outcomes. In contrast, modern approaches leverage technology to achieve greater precision and efficiency. Advancement in technology has shifted to a new dimension of predictive and monitoring in cattle health management. This review aims at highlighting the available and current digital technologies in cattle health, evaluate their utility in practice, and identify possible future advancements in the field that can potentially bring even more changes to this industry. The paper highlights some of the barriers and disadvantages of using these technologies, such as data security issues, high capital investments, and skills gap. The integration of these advanced technologies is set to play a fundamental role in enabling the livestock industry to meet the rising global demand for high-quality, sustainably produced products. These technologies are essential for ensuring compliance with ethical standards and best practices in cattle care and well-being. In light of these advancements, the application of digital innovations will support the achievement of socially responsible cattle production, while simultaneously maintaining optimal levels of animal health and welfare.

## 1 Introduction to digital tools in cattle health

A key goal of livestock rearing is to ensure optimal health and welfare of animals, not only for ethical and economic reasons, but also to meet increasing societal demands for products that are originated from animals with high welfare standards, i.e., raised under conditions where they can thrive (1, 2). Digital technologies that quantify aspects of animal behavior, physiology, and production over time have the potential to contribute to the identification of welfare and health related problems early on (3).

This in turn can enable a rapid intervention at herd or individual level to improve the animals’ living conditions, prevent animals from suffering, improve treatment efficacy and reduce antibiotic consumption (4, 5). This mini-review provides an overview of the most common digital tools currently used within the cattle sector where the integration digital tools has led to enhanced monitoring capabilities, allowing farmers to collect real-time data on cattle health and behaviors. By utilizing technologies such as wearable sensors, drones, and mobile applications, stakeholders can make informed decisions that promote better welfare outcomes and disease prevention strategies. Digital tools have led to practical improvements for both researchers, the cattle industry, and veterinarians.

**Figure 1:**
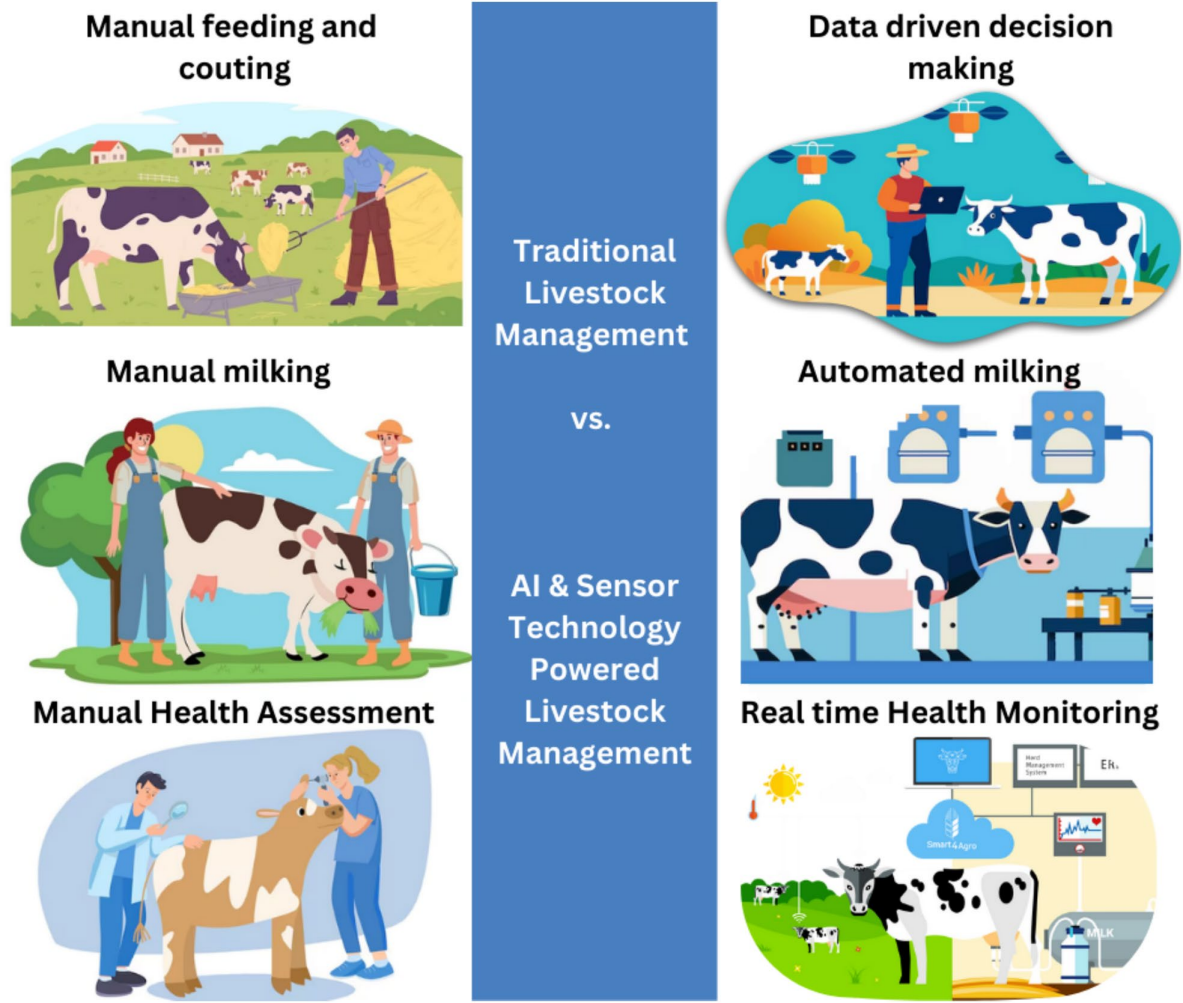
From tradition to precision in cattle farming.

The use and market penetration of digital technologies for health management within the cattle sector is considered relatively low in some countries when compared to other sectors such as pig or poultry farms. Initially, digital tools in the cattle sector were mainly introduced as an aid to both monitoring production quantities and quality. Indeed, the need to support management decisions on a range of aspects such as feeding, heat detection, the timing of insemination, and the knowledge around the different growth curves created a demand for data collection tools (3, 6) (Figure 1).

At a herd level, management tools now allow for automated data collection and herd management, outfitted with data collection on production details, such as yields and compositions, for all cows. In certain nations, the implementation of data management tools differs depending on the respective industry sector (7). For example, France and Ireland demonstrate significantly contrasting proportions of dairy farms employing cow identification, monitoring milk production peaks, and engaging with a milk advisor. Recent studies have also documented that specific digital tools have facilitated optimization of labor productivity, logistics, and operations, or have improved the monitoring of technical data (8, 9).

## 2 Practical applications of digital technologies in cattle management—case studies

Animal-friendly cattle management strategies, which include providing the best possible conditions for the health, welfare, and productivity of cattle, are key factors in achieving sustainability in cattle production systems (10, 11). In this context, the use of digital technology in the field of cattle management can facilitate more effective and efficient preventive as well as curative animal care. These applications encompass both on-animal devices and sensors embedded within the living environment, which monitor the behavior, breeding, and health of cattle (12, 13).

Traditionally, the animals’ behavior and health are controlled by visual observation and handling, and are frequently influenced by treatment. As the livestock sector is evolving, the farmers’ opportunities to work with the herd and the animal caretaker’s activity periods must also be considered (14). Given the forecasted shortage of 64,000 workers by 2028 that the dairy sector alone faces, as well as the urgent need to focus on both improving farm efficiency and increasing animal health and welfare, there is an ongoing need to develop technologies that are science-based for ensuring minimal disturbance of the animals, as well as a better and more efficient use of resources. Similarly, concerns about the safety and quality of dairy products have led to development for innovative methods and tools of assessment (15, 16).

Recent advancements in AI, IoT, and sensor technologies have improved cattle health monitoring, disease prevention, and farm management. Their impact is demonstrated through several case studies carried out over the past years. One such study was conducted by Marku et al. (17) who explored the implications of digitalization in livestock farming in two distinct settings: Baden-Württemberg (Germany), respectively North Savonia (Finland). Farmers in Finland, for example, perceive digitalization as an essential catalyst in boosting collaboration with industry partners, facilitating access to new markets, and optimizing financial resource allocation. In contrast, German farmers were less confident of its transformative impact on these elements, emphasizing regional differences in digital usage. These findings highlight the importance of personalized digital approaches that address farm-specific and regional difficulties in order to fully reap the benefits of precision livestock production.

Digi4Live, a Horizon Europe project spanning 2024–2028, exemplifies the multinational efforts to advance digital livestock technologies (18). The initiative, which includes 16 partners from nine countries, intends to boost digital technology adoption in the European cattle sector, benefiting farmers, agribusinesses, and policymakers. The effort aims to create over 50 data-driven cattle management solutions using embedded sensors, AI-powered computer vision, and IoT technologies. Digi4Live has pioneered two key technologies for cattle monitoring. The first one is Computer Vision for Behavior Analysis, a technology that automates behavior tracking, enhancing animal welfare assessment. GPS Sensors for Dairy Cows, is the second solution developed, designed to monitor outdoor access and grazing patterns, thus improving sustainability and resource efficiency. To strengthen AI-based animal tracking, the project integrates multi-farm datasets from 20 dairy farms with diverse environmental conditions. A novel web-based tool, called Smart Labeling Loop, that facilitates manual data labeling to train neural networks, ensuring robust algorithm development will also be developed. The project’s goal is to demonstrate the reliability of digital tracking technologies across different farm settings, refine prediction models for outdoor access, and establish general guidelines for AI-driven monitoring systems (18).

Beyond improving livestock monitoring, Precision Livestock Farming (PLF) technologies are also recognized as an effective strategy for greenhouse gas (GHG) mitigation. In a study conducted by (49), data from the Scottish Cattle Tracing System (CTS) was used to model the impact of PLF adoption on carbon emissions in beef production systems. The research team developed two baseline scenarios—one for grazing systems and one for housed systems—and calculated emissions using the Agrecalc carbon footprinting tool. They analyzed the effects of automatic weigh platforms, accelerometer-based estrus detection sensors (fertility sensors), and health sensors for early disease detection on farm-level emissions. The findings indicated that PLF adoption had a greater impact in housed systems than in grazing systems, suggesting that technology-driven efficiency improvements could significantly reduce the carbon footprint of beef production. Although this study focused on Scotland, it is likely that similar emission reductions can be achieved in other European countries with comparable farming practices.

The use of PLF sensors represents an essential mechanism to reduce how climate change affects dairy cattle welfare. The research by Ranzato et al. (19) evaluated behavioral adaptations of Italian Holstein cows under heat stress conditions on a precision livestock farming facility. The dairy farm experienced three hot weather events defined as heat waves that lasted for five straight days where the temperature-humidity index exceeded 72 in the summer of 2021. A study with 102 cows studied milk yield records to identify animals that showed reduction in milk without concurrent mastitis symptoms. The ear-tag-based accelerometer sensors tracked both the time spent laying down and chewing action alongside total movement patterns. Results from the research revealed heat waves prompt all cows to chew more often and move around more frequently during the day while shortening the time they spend resting. Heat-sensitive animals spent 15 more daily minutes carrying out these activities. Device-generated frequent sensor data enables accurate identification of cows requiring specific heat stress relief methods resulting in better animal welfare outcomes along with production gains.

## 3 Future developments and innovations in digital tools for cattle health

The constant improvement of technological innovation in recent decades is providing a solid and competitive base for the “fourth agricultural revolution,” a “digitally augmented era” which is expected in the coming years (9). In animal production, different digital technologies, in particular the Internet of Things, big data, robotics, and artificial intelligence, provide the basis to originate the so called Precision Livestock Farming (Table 1). This allows for the continuous collection of an ever-increasing amount of data of direct interest, which may be sent and processed so that new decisions may be made in a positive feedback loop (20).

**Table 1:** Applications of digital technologies in cattle management.

Domain of application	Research developments	Benefits	Challenges	References
Health, reproduction, feeding and behavior monitoring	Wearable devices track health and feeding data—e.g., movement activity, feed intake, frequency, duration—in real-time using IoT-enabled sensors. Technologies such as bioacoustics, accelerometers, infrared thermography are also employed for collection of data. Using artificial intelligence, deep learning, cloud-based systems analyze data to identify anomalies and provide farmers practical insights. Predictive analytics research is being conducted to foresee changes in feeding habit caused by health or environmental conditions.	Early detection of diseases or stress through real-time monitoring, reducing mortality rates and improving animal welfare. Optimized feeding strategies based on individual animal needs, enhancing growth efficiency and reducing feed waste. Behavioral insights help identify issues like lameness or aggression, enabling timely interventions.	High initial costs for sensors, cameras, and data management systems. Data overload can overwhelm farmers without proper training or analytical tools. Wearable devices may cause discomfort or require frequent maintenance.	(19, 30–34)
Non-invasive weight assessment	Automated systems use imaging technologies, load cells, or 3D cameras to estimate animal weight without physical handling. This reduces stress on animals and provides continuous growth data, helping farmers adjust feeding regimes and predict market readiness.	Non-invasive weight measurement reduces stress on animals and labor for farmers. Continuous growth tracking allows for precise feeding adjustments and better market timing. Improves breeding and selection processes by providing accurate performance data.	Accuracy can be affected by animal movement or environmental factors. High upfront costs for imaging systems or load cells. Requires calibration and technical expertise to ensure reliable data.	(35–37)
Environmental monitoring	IoT-enabled sensors to monitor environmental parameters such as temperature, humidity, air quality, and ammonia levels. Automated ventilation, misting and heating systems can be adjusted based on real-time data.	Ensures optimal living conditions, improving animal health and productivity, especially in the context of heat stress Reduces energy costs by automating ventilation, heating, and cooling systems. Minimizes environmental impact by controlling emissions like ammonia and methane.	Sensors and IoT devices require regular maintenance and calibration. Data integration from multiple sensors can be complex. Initial setup costs may be prohibitive for small-scale farmers	(38–40)
Traceability of the dairy and beef supply chain	RFID and GPS technologies are widely adopted for tracking livestock movement across the supply chain. AI-powered analytics predict bottlenecks and optimize logistics, ensuring timely delivery and animal welfare. Research focuses on blockchain integration for enhanced traceability and transparency. AI models are being developed to predict supply chain disruptions and optimize inventory management.	Enhances food safety by enabling quick identification of contamination sources. Builds consumer trust through transparency in product origins and handling. Improves supply chain efficiency by reducing losses and streamlining logistics.	Implementing blockchain or RFID systems requires significant investment. Data privacy and security concerns may arise. Small-scale producers may struggle to adopt these technologies due to cost and complexity.	(41–44)
Market dynamic analysis	AI analyzes market trends, demand–supply dynamics, and price fluctuations, providing predictive insights for production and exports. IoT sensors collect data at various supply chain stages for real-time market analysis. Research is advancing in AI models for more accurate market predictions, including consumer preferences and global trade trends. Integration of AI with blockchain for secure and transparent market data sharing.	Enhances food safety by enabling quick identification of contamination sources. Builds consumer trust through transparency in product origins and handling. Improves supply chain efficiency by reducing losses and streamlining logistics.	Implementing blockchain or RFID systems requires significant investment. Data privacy and security concerns may arise. Small-scale producers may struggle to adopt these technologies due to cost and complexity.	(45–48)

The expanded capabilities of sensors and data processing, together with advances associated with cloud computing, fast analytical algorithms, big data, and machine learning, are enabling ambitious and complex systems to be developed not only for highly specialized applications in different contexts like smart farming agriculture-oriented, but also for the smart livestock tasks (21). When it comes to cattle, possibly the best-known application of Internet of Things tools is the automatic milking system (22). Technologies for milk production monitoring include, in addition to equipment for milking, radiofrequency identification ear tags and collars, as well as accelerometer-based devices that monitor the activity and rumination of individual animals (23, 24).

These tools are either utilized as separate devices on individual animals or combined together to complement the data in the “Integrated System” of production, in a farming automation context, very close to the Internet of Living Things vision. Over the past years, various IoT tools that provide risk alerts to improve on-farm control of animal welfare, allowing for the early detection of diseases like respiratory infections, nutritional alterations, mastitis or physiological modifications such as calving, or estrus have been developed (25).

## 4 Challenges and barriers to implementing digital technologies in cattle production

In order to effectively deploy digital technologies across a broad spectrum of cattle producers and related industries like those related to health and welfare, it is important to appreciate and address barriers to scaling that deployment. This is specifically important, as animal welfare is ultimately the responsibility of the owner and the manager of the animal, regardless of the level of digital assistance (26). There are many different types of cattle production systems, but not all of these systems are applicable to every single production context. For example, high-input outdoor grass-fattened cattle operations will face very different issues and require varying resources than low-input extensively managed animals do.

Additionally, these concerns are distinct from those encountered in feedyards, where conditions and management strategies vary significantly. Each type of production has its own unique set of challenges and advantages, tailored to its specific approach in the cattle industry (27). The aim of cattle production and the associated challenges are very different in these settings. Producers are motivated by many different factors to adopt technology. Ultimately, those products that can improve efficiency or overall economics are needed. Access to cost-effective technology is also a critical limit to many adoption issues (7, 28). Products that do not pay for themselves in a reasonable amount of time may not be adopted. Since not all production practices are alike, the potential economic benefits of a digital technology may not apply to everyone, which limits the target audience.

The digital divide, that is, a gap in labor skill or access to technology, might also limit the number of users (29). Additional training or troubleshooting may be required when implementing new technologies, especially in environments where the use of technologies does not occur regularly. A perceived lack of value to the producer might also reduce willingness to invest, even if the product is now affordable. Returns and overall positive outcomes are easier to demonstrate to potential buyers if supported by data; while product manufacturers may provide data, they may also be biased. Data ownership and data rights are very important, as the data may be leveraged in both the short term and long term (28). It is also not uncommon for the potential user to have several reasons why adoption would not be beneficial to them, which is what extension and academia are trying to uncover, understand, and ameliorate.

Research is important to identify barriers, including actual technology adoptions. Qualitative data can also provide insights into motivations and actions for each specific region, since the concept of one size fits all, may not be applied to all contexts.

## 5 Conclusion

The future is bright, bearing in mind predictions regarding the advances in artificial intelligence over the coming years. This could take the form of more sophisticated image analysis that is able to monitor more traits of interest. Developments in sensor technology in terms of miniaturization and computing capability are also expected to assist with this. For those technologies that are currently emerging, such as the deployment of wearable sensors for calf monitoring, opportunities will need to be identified for how and where these systems are adopted.

Many of these digital technologies are underpinned by artificial intelligence or machine learning. Collectively, the application of digital technologies is seen to improve economic productivity, including through improved animal health, welfare, and environmental sustainability. A common thread throughout the subsections was the role of digital technologies not just as a tool but as a driver to deliver sustainable changes to beef and dairy farming. The uptake of digital technologies is delivering significant data and information for management analysis, environmental assessment, managing carbon emissions, feeding practices, and the delivery of new consumer-facing options. Although the adoption of digital technologies is accelerating at an unprecedented pace, many of the present findings not yet reflect the leading edge of development.

Ongoing practical application and adaptive research, aimed at policy and practice application, must be encouraged to inform regarding the development and uptake of digital technologies. These will further encourage a mutually beneficial engagement between the cattle industries, associated stakeholders, and the digital technology sector. It is evident that cattle farming practices of the future will require substantial multi-industry, cross-institutional decision-making and engagement. It is both an exciting time and opportunity for the livestock monitoring and digital technology industries. While the deepening and implementation of this knowledge and use of digital technologies will benefit the economy and social well-being, it will also benefit the global cattle industry, our ecosystems, and the outputs of associated support industries.
